# MiR-191-5*p* is upregulated in culture media of implanted human embryo on day fifth of development

**DOI:** 10.1186/s12958-021-00786-1

**Published:** 2021-07-13

**Authors:** Ricardo Josué Acuña-González, Mercedes Olvera-Valencia, Jorge Skiold López-Canales, Jair Lozano-Cuenca, Mauricio Osorio-Caballero, Héctor Flores-Herrera

**Affiliations:** 1 Department of Immunobioquímica, Instituto Nacional de Perinatología “Isidro Espinosa de los Reyes” (INPerIER), Ciudad de México, México; 2 Department of Biología de la Reproducción, INPerIER, Ciudad de México, México; 3 Department of Fisiología y Desarrollo Celular, INPerIER, Ciudad de México, México; 4 Department of Salud Sexual y Reproductiva, INPerIER, Ciudad de México, México

**Keywords:** MiRNA expression, Embryo development, Implantation, Embryo culture media, *In vitro* fertilization

## Abstract

**Background:**

Morphological features are the most common criteria used to select human embryos for transfer to a receptive uterine cavity. However, such characteristics are not valid for embryos in cellular arrest. Even aneuploid embryos can have normal morphology, and some euploid embryos have aberrant morphology. The aim of this study was to quantify the expression profile of hsa-miR-21-3*p*, -24-1-5*p*, -191-5*p*, and -372-5*p* in culture media on day 5 of *in vitro* embryo development, and compare the profiles of two groups of media classified by outcome: successful (n = 25) or unsuccessful (n = 25) implantation pregnancy.

**Methods:**

Fifty patients were accepted in the Department of Reproductive Biology of a Hospital in México City, based on the Institutional inclusion criteria for *in vitro* fertilization. Embryos were transferred to the women on day 5 of cultivation, and the culture media were collected. RNA was isolated from each culture medium with TRIzol reagent, and microRNA (miRNA) expression was detected through RT-PCR with specific primers. Expression bands were quantified by reading optical density.

**Results:**

There was a 5.2-fold greater expression of hsa-miR-191-5*p* in the pregnancy-related culture media (*p* ≤ 0.001) and a 1.6-fold greater level of hsa-miR-24-1-5*p* (*p* = 0.043) in the media corresponding to non-pregnant women. No significant difference existed between the two groups hsa-miR-21-3*p* (*p* = 0.38) or hsa-miR-372-5*p* (*p* = 0.41).

**Conclusions:**

Regarding adequate *in vitro* embryo development, hsa-miR-191-5*p* could possibly represent a positive biomarker, while has-miR-24-1-5*p* may indicate poor prognosis. This former miRNA modulates IGF2BP-1 and IGF2R, associated with the implantation window. On the other hand, hsa-miR-24-1-5*p* may be related to a poor prognosis of human embryo development.

## Background

MicroRNAs (miRNAs) are a large class of small non-coding RNAs approximately 23 nucleotides in length. Their important functions in post-transcriptional gene expression are carried out by binding to the complementary sequence of the 3′untranslated region (3′-UTR) or by degrading target messenger RNA (mRNA) transcripts via complementary base pairing [1]. MiRNAs are essential to many cellular processes and can be transferred between cells as a mode of cell-cell communication [2] and are relatively stable when circulating [3].

MiRNAs regulate cell differentiation and proliferation [4], apoptosis [5], endometrial receptivity [6], and decidualization [7]. Moreover, they are involved in the pathogenesis of some diseases, including endometrial cancer [8, 9]. Deficiencies in the processing of pre-miRNAs are associated with defects in embryonic development [10, 11]. Capalbo et al. (2016) made an analysis of miRNAs in the culture medium of developing embryos, finding an expression profile similar to that of trophectoderm cells [12]. According to several reports, various miRNAs are probably involved in embryo development. While miR-21 has potent anti-apoptotic activity in ovarian granulosa cells [13, 14], and participates in the development of primordial follicles [15], and the maturation oocytes [16, 17]. MiR-24 has been linked to fertilization, reproductive processes and the implantation window in experimental models [18–20]. Additionally, miR-191 and miR-372 have been detected in human embryonic stem cells [21] and the initial stage of implantation [22]. They are involved in trophoblast proliferation and invasion signal pathways, such as STAT3/ERK1 [17, 23], AKT/ERK1/MAPK [24], and SMAD [25].

The aim of the current study was to quantify the expression profile of hsa-miR-21-3*p*, -24-1-5*p*, -191-5*p*, and -372-5*p* in culture media on day 5 *in vitro* fertilization (IVF) embryo development, and compare the profiles of two groups pf media classified by outcome: successful (n = 25), or unsuccessful (n = 25) implantation and pregnancy in patients.

## Methods

### Ethics approval

Embryos were cultured in media and transferred to women on the fifth day of development. On this same day, the media were collected for analysis. The current protocol was approved by the Ethics and Research Committees of the Instituto Nacional de Perinatología, Ciudad de México, México (212250-22661). The prospective participants received an explanation of the purpose of the present investigation, and those willing to take part signed informed consent. All candidates signed the consent form.

### Patient selection

The study was conducted on a total of 50 female patients who received a diagnosis of infertility and requested *in vitro* fertilization (IVF). The inclusion criteria were the following: ≤37 years of age, regular menstrual cycles, normal uterine cavity confirmed by hysteroscopy, the absence of intrauterine adhesion or inflammation, ≥7 mm endometrial thickness in the late follicular phase (determined by ultrasonography), a normal ovarian reserve (<9.0 mU/mL follicle-stimulating hormone), a normal ovarian response to the stimulation protocols (>8 oocytes retrieved in a controlled ovary hyperstimulation cycle), and no use of exogenous hormone (estradiol/progesterone) during the endometrial cycle. The exclusion criterion was the failure of the woman to undergo an ultrasound scan within 4 weeks after a positive pregnancy test. Non-inclusion criteria were endometrial cancer or hyperplasia, endometriosis, and having a male partner with infertility.

### Ovarian stimulation

The patients received controlled ovarian stimulation based on an assessment of FSH/LH. Upon observing a follicular cell greater than 18 mm in diameter, the ovaries were stimulated with chorionic gonadotropin (hCG, Merck Serono, Switzerland). Mature oocyte were captured with ultrasound guidance 36 h posterior to hCG injection, and IVF was performed on day 5 after oocyte retrieval.

### In vitro fertilization

Fertilization of oocytes was carried out *in vitro* with capacitated sperm (1 × 10^6^ cells/mL) and evaluated by the presence of a second polar body. The progression of cell division was monitored daily until it reached the 36-cell stage. Successfully fertilized oocytes were individually maintained in 25 μL of G-1 PLUS culture medium (Vitrolife Sweden AB, Sweden). The embryos were cultured in a ASTEC incubator (EC6S-MD, USA, PA) at 37 °C with 5% O_2_/6% CO_2_ until being transferred to women on day 5.

Two embryos with type I, II, or III quality on the third day of embryonic development were transferred to the uterine cavity during the implantation window using the Soft Cook technique and Flexible Pass intrauterine transfer cannula guided by an abdominal ultrasound apparatus equipped with a real-time, 5-MHz sector electronic array endovaginal probe (Philips Epiq CVx; MO, USA).

Fourteen days after embryo transfer, ultrasound was employed to examine the possibility of successful implantation, evidenced by the development of the embryonic sac. Based on the results, the patients were assigned to one of two groups: (1) implanted embryos (n = 25, pregnant patients) and (2) non-implanted embryos (n = 25, non-pregnant patients).

### RNA isolation, cDNA synthesis, and PCR-reaction

The culture medium was obtained on day 5 of embryonic development, and total RNA was extracted with the TRIzol reagent (InvitroGen, Carlsbad, CA), according to the manufacturer´s instructions. The concentration of RNA in each sample was measured as the A_260_/A_280_ ratio on a NanoDrop One spectrophotometer (Thermo Scientific, Waltham, MA, USA).

The purified RNA was subjected to complementary DNA synthesis was carried out with AMV-Tfl (A1260, Madison WI, USA) as specified by the manufacturer’s. The total volume of the reaction mixture was 20 μL, consisting of 2 μL of an RNA samples, 5 μL buffer AMV-TfI 1X, 1 μL dNTP (10 mM), 2 μL MgSO_4_ [50 mM], 10 μL ddH_2_O, 1 μL AMV RT and 1 μL (20 pmol) of a specific sequence used as the primer. The followings were the primers: hsa-miR-21-3*p* (GTCGTATCCAGTGCAGGGTCCGAGGTATTCGCACTGGATACGACACAGCC), hsa-miR-24-1-5*p* (GTCGTATCCAGTGCAGGGTCCGAGGTATTCGCACTGGATACGACACTGAT), hsa-miR-191-5*p* (GTCGTATCCAGTGCAGGGTCCGAGGTATTCGCACTGGATACGACCAGCTG), and hsa-miR-372-5*p* (GTCGTATCCAGTGCAGGGTCCGAGGTATTCGCACTGGATACGACAGAATA). The reactions mixture was incubated at 45 °C for 45 min.

Three μL of cDNA product from the culture medium sample were amplified by PCR reactions, carried out in 0.2 mL PCR tubes in a thermocycler (Techne touchgene gradient). Each tube contained 5 μL of buffer AMV-Tfi 1X, 1 μL (10 mM) dNTP, 2 μL (50 mM) MgSO_4_, 10 μL ddH_2_O, and 1 μL (20 pmol) of a primer sequence. The followings were the primers: hsa-miR21-3*p* (CGGCCGCAACACCAGT), hsa-miR24-1-5*p* (CGGCCGTGCCTACTGA), hsa-miR-191-5*p* (CGGCCGCAACGGAATC), and hsa-miR-372-5*p* (CGGCCGCCTCAAATGTG). Additionally, 1 μL specific universal sequence (5′-GTG CAG GGT CCG AGG T-3′) was included to afford the hairpin structure that is instrumental for detection [26]. PCR cycling conditions were 94 °C for 30 s followed by 40 cycles (94 °C for 30 s, 56 °C for 30 s, 72 °C for 30 s) and finally 72 °C for 10 min.

Twenty μL of PCR amplicons were mixed with Tris/Acetic Acid EDTA 1X loading buffer (Bio-Rad, Hercules, CA, USA) and added to wells containing 4.0% agarose gel. Electrophoresis was then run at 60 V at a constant temperature for 40 min.

Subsequently, the gel in each well was visualized and the image captured by a UV transillumination system (Gel Doc 2000, Bio-Rad, Hercules, CA, USA). The band of expression for each miRNA of interest was identified by optical density with the ImageJ program (NIH, USA).

### Blood samples

Five milliliters of peripheral blood were obtained from the patients by puncture of the cephalic vein, placed in EDTA-K2 tubes (BD Vacutainer), and centrifuged at 14,000 rpm for 10 min. Serum was collected in Eppendorf tubes and stored at −70 °C until to quantification hormones assay.

### Assay to determine the levels of sex hormones

All evaluations of hormones were performed in the central laboratory of the Instituto Nacional de Perinatología on the Modular Analytical apparatus cobas e 411 (Roche, USA). A commercially available assay kit was used to measure the serum levels of P4, E2, T4, FSH, LH, AMH, and hCG (Roche system, USA), with according to the manufacturer´s recommendations and as previously described [27–29]. The lower limit of detection for these hormones was 0.4 ng/mL, 5 pg/mL, 0.025 pg/mL, 0.100 mIU/mL, 0.100 mIU/mL, and 0.2 ng/mL, 0.1 mIU/mL, respectively. The intra-assay coefficient of variation was 3%, 5%, 5%, 3%, 2%, 3%, and 5% respectively.

### Statistical analysis.

Significant differences between the two groups of women (those with implanted or non-implanted embryos) were determined in relation to patient characteristics, hormonal concentrations, and expression of miRNAs.

Data are expressed as the mean ± SD and examined with Student’s *t-*test on SigmaStat version 4.0 (Jandel Co., CA, USA), considering *p* < 0.05 as statistically significant.

## Results

### Patient characteristics

The clinical characteristics of pregnant and non-pregnant patients were show in Table 1. There were no significant differences between the two groups in regard to mean age (*p* = 0.23), body mass index (*p* = 0.43), length of the menstrual cycle (*p* = 0.71), duration of menstruation (*p* = 0.54), or endometrial thickness on the day of the LH surge (*p* = 0.45).

**Table 1:** Characteristics of the two groups of women undergoing endometrial receptivity those with implanted and non-implanted embryos

Variable	Implanted (n = 25)	Non-implanted (n = 25)	*p*-value
Age, (years)	35.7 ± 2.4	36.8 ± 3.1	0.23
BMI, (kg/m^2^)	27.2 ± 3.7	26.3 ± 3.2	0.43
Menstrual cycle length, (days)	27.1 ± 4.4	27.9 ± 4.9	0.71
Menstrual duration, (days)	5.1 ± 1.9	5.3 ± 1.4	0.54
Endometrial thickness, (cm)	10.2 ± 0.8	9.8 ± 0.9	0.45

*BMI* Body mass index. Data are presented as the mean ± standard deviation

### Hormone profiling

The concentration of hormone was compared between pregnant and non-pregnant patients (Table 2). No significant differences existed with respect to P4 on the day of final oocyte maturation (*p* = 0.51), E2 in the non-follicular phase (*p* = 0.88), E2 in the follicular phase (*p* = 0.21), T4 (*p* = 0.70), FSH (*p* = 0.34), LH (*p* = 0.72), or the Anti-Mullerian Hormone (*p* = 0.44). However, significant difference was indeed found for hCG (*p* < 0.001; Table 2).

**Table 2:** Comparison of the hormonal concentration between women with implanted and non-implanted embryos

Variable	Implanted (n = 25)	Non-implanted (n = 25)	*p*-value
P4 on day of final oocytes maturation, (ng/mL)	0.80 ± 0.34	0.74 ± 0.35	0.51
E2 no-follicular phase, (pg/mL)	60.1 ± 15.2	59.4 ± 18.5	0.88
E2 follicular phase, (pg/mL)	1636.0 ± 528.4	1399.4 ± 779.5	0.21
T4 follicular phase, (pg/mL)	53.0 ± 23.5	49.1 ± 26.7	0.70
FSH follicular phase, (mIU/mL)	5.8 ± 1.9	6.3 ± 1.8	0.34
LH follicular phase, (mIU/mL)	5.3 ± 1.6	5.4 ± 1.6	0.72
AMH, (ng/mL)	2.6 ± 1.1	2.4 ± 1.1	0.44
hCG, (mIU/mL)	4.2 ± 13.5	73.5 ± 20.9	≤0.001

Data are presented as the mean ± standard deviation

### Expression of the miRNAs of culture media

**Fig. 1:**
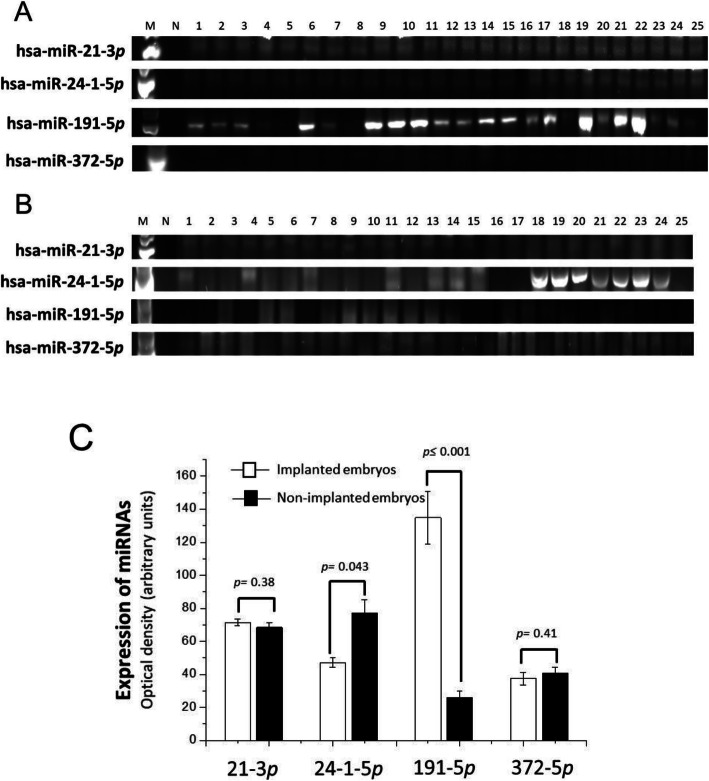
**Expression of hsa-miRNAs in the culture medium of human embryos with type II development**. Marker (Lane 1), negative control (Lane 2) samples of the culture medium of the 25 implanted (**A**, pregnant patients) and non-implanted embryos (**B** non-pregnant patients). The optical density of each band was determined and the mean ± standard deviation is shown (**C**). The significant difference is indicated which was made by the Student's t-test and was taken as a difference of less than 0.5

A comparison was made of the expression profiles of the two groups of culture media based on the optical density of each miRNA of interest (Fig. 1). The relative optical density detected in the pregnant versus non-pregnant patients was 71.5 ± 2.0 *vs* 65.56 ± 2.6 for has-miR-21-3*p*, 47.18 ± 2.9 *vs* 77.0 ± 8.3 for has-miR24-1-5*p,* 134.91 ± 15.91 *vs* 24.82 ± 4.3 for has-miR-191-5*p*, and 37.43 ± 3.8 *vs* 40.73 ± 3.7 for hsa-miR-372-5*p*. According, the most abundantly expressed miRNA in pregnant patients was hsa-miR-191-5*p* and the one with the lowest expression was has-miR-372-5*p* (Fig. 1C). In non-pregnant patients, hsa-miR-24-1-5*p* had the expression and hsa-miR191-5*p* the least (Fig. 1C). The comparison between groups showed a 5.2-fold higher level for hsa-miR191-5*p* in the culture media embryos that led to pregnancy (*p* ≤ 0.001), and a 1.6-fold higher level for hsa-miR-24-1-5*p* in the culture media embryos that were not successfully implanted (*p* = 0.043). There were no significant differences in relation to hsa-miR-24-1-5*p* (*p* = 0.38) or hsa-miR-372-5*p* (*p* = 0.41; Fig. 1C).

## Discussion

Although embryonic morphology is the criterion used in clinical practice to evaluate the viability of embryos for transfer to patients [30, 31], this method selection has not demonstrated an acceptable correlation, with outcome. Even aneuploid embryos can have normal morphology, and some euploid embryos can have aberrant morphology [32]. Among the various proteins reported as possible biomarkers for embryonic development [31, 33], none have yet proven to be good indicators of a successful implantation in the receptive endometrium.

During the development of murine embryos from the first stages of cell division to the formation of blastocysts, expression of miRNAs predominates over other non-coding RNAs, suggesting their likely role in regulating distinct pathways of differentiation and cell proliferation [34]. In the current study, the comparation of the expression of four miRNAs between the two groups of embryo culture media (corresponding to pregnant versus non-pregnant patients) revealed important differences. There was a stronger expression of hsa-miR-191-5*p* in the culture media of embryos leading to pregnancy (*p* ≤ 0.001), and of hsa-miR-24-1-5*p* in the samples corresponding to non-pregnant patients (*p* = 0.043). The miRNAs that showed no significant difference between two groups were hsa-miR-21-3*p* and hsa-miR-372-5*p* (Fig. 1C).

**Fig. 2:**
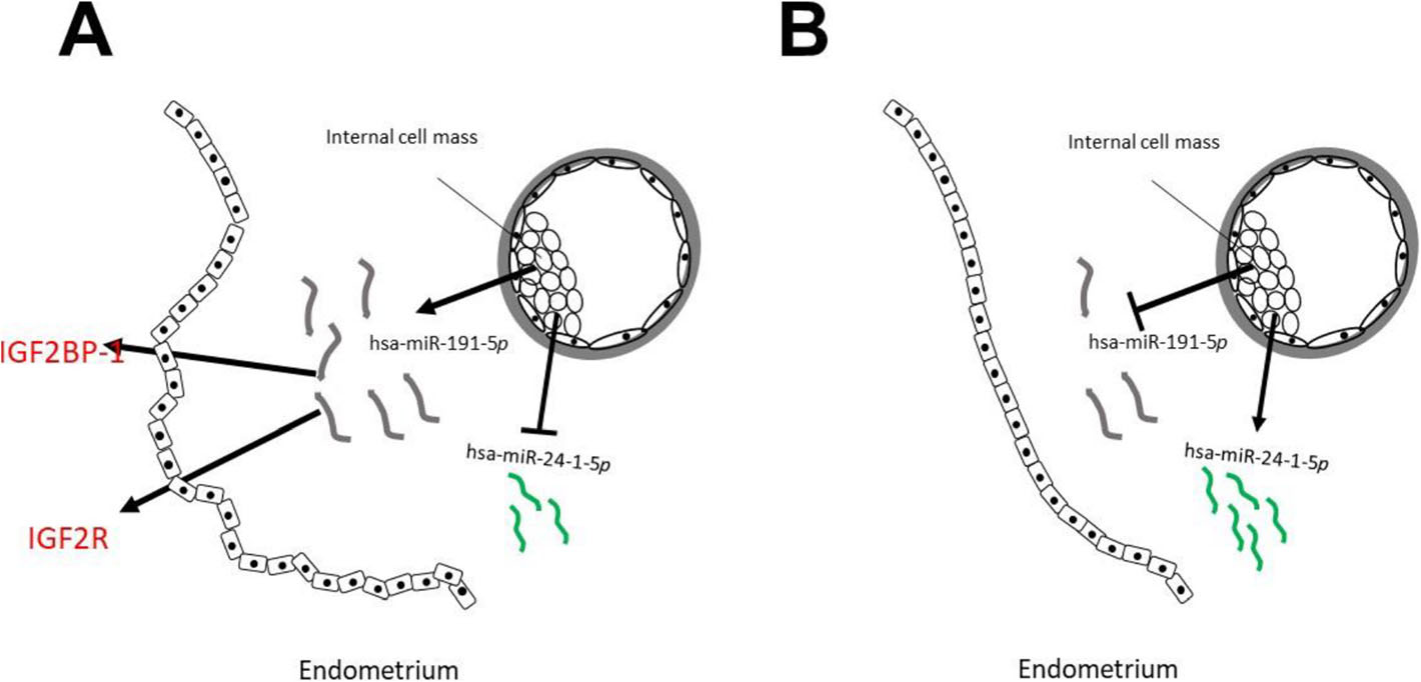
**Differential expression of hsa-miR-191-5*p*** **and hsa-miR-24-1-5*p*** **in the culture medium of human embryos with type II development**. The secretion of hsa-miR-191-5*p* by developing embryos stimulates the expression of insulin-like growth factor-associated proteins (IGF2BP-1 and IGF2R) associated with the implantation window in endometrial cells and responsible for inducing major changes in the decidualization of endometrial tissue (**A**) [14]. For its part, the increase in the expression of hsa-miR-24-1-5*p* in the culture medium of developing embryos of non-implanted (non-pregnant patients) has been associated with inhibition in cell proliferation and migration (**B**)

The activity of hsa-miR191-5*p* on endometrial markers in the implantation window and of hsa-miR-24-1-5*p* on cell proliferation and migration is represented in a model (Fig. 2). Rosenbluth *et al.,* (2014) described an increase in miR-191 in the culture media of developing embryos having undergone implantation [35]. The current results indicate a significant 5.2-fold greater expression of this miRNA in the culture media of embryos of pregnant versus non-pregnant patients. Recently Wang *et al.,* (2016) reported that the expression of hsa-miR-191-5*p* modulates various proteins, two of which belong to the insulin-type growth factor family (IGF2BP-1 and IGF2R), associated with the decidualization of endometrial tissue [36]. According to the data in the literature, miRNAs, are not only potential biomarkers of implantation feasibility, but also could be secreted to the extracellular environment to induce activation of the cells or white tissues favoring embryo implantation and embryonic development.

Kropp *et al.,* (2015) transfected morula stage embryos with a mimic of miR-24. When they progressed to the blastocyst stage, there was morphological damage (compared to the control group), apparently related the activation of cellular apoptosis [20]. The authors demonstrated that one of the target mRNAs of miR-24 is *CDKN1b*, a cell cycle regulator found to be significantly reduced in the experimental control group [20]. Moreover, an elevated expression of hsa-miR-24 has been linked to the suppression of osteoblast differentiation [37], failure of embryo implantation into the endometrium [38], and adverse obstetrics tests results related to preeclampsia and intrauterine growth restriction [38, 39]. Thus, the current 1.6-fold greater expression of has-miR-24-5-*p* in the culture media of embryo of non-pregnant versus pregnant patients is in agreement with previous reports (Fig. 1C).

The two miRNAs with a lack of significant difference between groups in the present study hsa-miR-21-3*p* and hsa-miR-372-5*p* are both “constitutive”, according to an analysis by prediction software. They are involved in the regulation of MAP3K-1 and CDK6 cyclin, which are critical genes in the cell cycle as well as the signaling and apoptosis pathways [40]. MiR-372-5*p* is known to aid in the conversion of human fibroblasts into pluripotential stem cells, suggesting a crucial role of this miRNA in balancing differentiation and the maintenance of cell pluripotency.

Consequently, some miRNAs are linked to embryonic viability and others to poor prognosis. In the future, the full description of embryonic miRNome may be an especially useful tool in the clinical field.

## Conclusions

The was a 5.2-fold greater expression of has-miR191-5*p* in the culture media corresponding to pregnancy and a 1.6-fold higher level of has-miR-24-1-5*p* in the media related to non-pregnant patients. Hsa-miR-191-5*p* could possibly serve as an invaluable biomarker of human embryo development, while has-miR-24-1-5*p* may be an indicator of poor prognosis of the same. The former miRNA modulates two proteins, IGF2BP-1 and IGF2R, to be associated with the implantation window. Our research group is currently evaluating the participation of has-miR-191-5*p* in the regulation of proteins associated with embryo implantation and in the activation of other miRNAs after the transfection of endometrial cells. In the future, the full description of the embryonic miRNA genome might be a very useful tool in the clinical field.

## Data Availability

All of the relevant information from the study is described in the manuscript.

## Abbreviations

BMI: body mass index
CDK6: cyclin-dependent kinase 6
IVF: in vitro fertilization
hsa-miR: homo sapiens-microRNA
PCR: polymerase chain reaction
RT: retrotranscription
MAP3K-1: mitogen-activated protein kinase

## References

[1] Wilczynska A, Bushell M (2015). The complexity of miRNA-mediated repression. Cell Death Differ.

[2] Valadi H, Ekstrom K, Bossios A, Sjostrand M, Lee JJ, Lotvall JO (2007). Exosome-mediated transfer of mRNAs and microRNAs is a novel mechanism of genetic exchange between cells. Nat Cell Biol.

[3] Turchinovich A, Weiz L, Langheinz A, Burwinkel B (2011). Characterization of extracellular circulating microRNA. Nucleic Acids Res.

[4] Li J, Wang G, Jiang J, Zhang L, Zhou P, Ren H. MicroRNA-127-3p regulates myoblast proliferation by targeting Sept7. Biotechnol Lett. 2020;42(9):1633–44. 10.1007/s10529-020-02906-0. Epub 2020 May 7. 10.1007/s10529-020-02906-0 32382971

[5] Hui P, Wang Y, Chen B, Wang Z, Qin S (2020). Mir-29c expression in glioma and its effects on tumor cell proliferation and apoptosis. Iran J Public Health.

[6] Altmae S, Martinez-Conejero JA, Esteban FJ, Ruiz-Alonso M, Stavreus-Evers A, Horcajadas JA, Salumets A (2013). MicroRNAs miR-30b, miR-30d, and miR-494 regulate human endometrial receptivity. Reprod Sci.

[7] Estella C, Herrer I, Moreno-Moya JM, Quinonero A, Martinez S, Pellicer A (2012). Simon C: miRNA signature and Dicer requirement during human endometrial stromal decidualization in vitro. PLoS One.

[8] Buchynska LG, Borykun TV, Iurchenko NP, Nespryadko SV, Nesina IP (2020). Expression of microRNA in tumor cells of endmetrioid carcinoma of endometrium. Exp Oncol.

[9] Zmarzly N, Hermyt E, Kruszniewska-Rajs C, Gola J, Witek A, Mazurek U, et al. Expression profile of EMT-related genes and miRNAs involved in signal transduction via the Wnt pathway and cadherins in endometrial cancer. Curr Pharm Biotechnol. 2020. 10.2174/1389201021666201218125900. 10.2174/1389201021666201218125900 33342410

[10] Boren T, Xiong Y, Hakam A, Wenham R, Apte S, Wei Z, Kamath S, Chen DT, Dressman H, Lancaster JM (2008). MicroRNAs and their target messenger RNAs associated with endometrial carcinogenesis. Gynecol Oncol.

[11] Yang WJ, Yang DD, Na S, Sandusky GE, Zhang Q, Zhao G (2005). Dicer is required for embryonic angiogenesis during mouse development. J Biol Chem.

[12] Capalbo A, Ubaldi FM, Cimadomo D, Noli L, Khalaf Y, Farcomeni A, Ilic D, Rienzi L (2016). MicroRNAs in spent blastocyst culture medium are derived from trophectoderm cells and can be explored for human embryo reproductive competence assessment. Fertil Steril.

[13] Gong J, Xu X, Zhang X, Zhou Y (2020). Circular RNA-9119 suppresses in ovarian cancer cell viability via targeting the microRNA-21-5p-PTEN-Akt pathway. Aging (Albany NY).

[14] Zhang X, Chen Y, Yang M, Shang J, Xu Y, Zhang L, Wu X, Ding Y, Liu Y, Chu M, Yin Z (2020). MiR-21-5p actions at the Smad7 gene during pig ovarian granulosa cell apoptosis. Anim Reprod Sci.

[15] Yao N, Lu CL, Zhao JJ, Xia HF, Sun DG, Shi XQ, Wang C, Li D, Cui Y, Ma X (2009). A network of miRNAs expressed in the ovary are regulated by FSH. Front Biosci (Landmark Ed).

[16] Bartolucci AF, Uliasz T, Peluso JJ (2020). MicroRNA-21 as a regulator of human cumulus cell viability and its potential influence on the developmental potential of the oocyte. Biol Reprod.

[17] Tscherner A, Brown AC, Stalker L, Kao J, Dufort I, Sirard MA, LaMarre J (2018). STAT3 signaling stimulates miR-21 expression in bovine cumulus cells during in vitro oocyte maturation. Sci Rep.

[18] Azizi E, Ghaffari Novin M, Naji M, Amidi F, Shams Mofarahe Z (2020). Does in vitro fertilization affect the expression of miRNAs and their biogenesis pathway in preimplantation mouse embryos?. Birth Defects Res.

[19] Cai H, Zhu XX, Li ZF, Zhu YP, Lang JH (2018). MicroRNA dysregulation and steroid hormone receptor expression in uterine tissues of rats with endometriosis during the implantation window. Chin Med J (Engl).

[20] Kropp J, Khatib H (2015). Characterization of microRNA in bovine in vitro culture media associated with embryo quality and development. J Dairy Sci.

[21] Paul ABM, Sadek ST, Mahesan AM (2019). The role of microRNAs in human embryo implantation: a review. J Assist Reprod Genet.

[22] Parks JC, McCallie BR, Patton AL, Al-Safi ZA, Polotsky AJ, Griffin DK, Schoolcraft WB, Katz-Jaffe MG (2018). The impact of infertility diagnosis on embryo-endometrial dialogue. Reproduction.

[23] Pereira de Sousa FL, Chaiwangyen W, Morales-Prieto DM, Ospina-Prieto S, Weber M, Photini SM, Sass N, Daher S, Schleussner E, Markert UR (2017). Involvement of STAT1 in proliferation and invasiveness of trophoblastic cells. Reprod Biol.

[24] Lim W, Song G (2016). Stimulatory effects of coumestrol on embryonic and fetal development through AKT and ERK1/2 MAPK signal transduction. J Cell Physiol.

[25] Kim KH, Kim EY, Kim GJ, Ko JJ, Cha KY, Koong MK, Lee KA (2020). Human placenta-derived mesenchymal stem cells stimulate ovarian function via miR-145 and bone morphogenetic protein signaling in aged rats. Stem Cell Res Ther.

[26] Wang GL, Zhang CY (2012). Sensitive detection of microRNAs with hairpin probe-based circular exponential amplification assay. Anal Chem.

[27] Baker VL, Rone HM, Pasta DJ, Nelson HP, Gvakharia M, Adamson GD (2006). Correlation of thyroid stimulating hormone (TSH) level with pregnancy outcome in women undergoing in vitro fertilization. Am J Obstet Gynecol.

[28] Csemiczky G, Wramsby H, Landgren BM (1996). Luteal phase oestradiol and progesterone levels are stronger predictors than follicular phase follicle stimulating hormone for the outcome of in-vitro fertilization treatment in women with tubal infertility. Hum Reprod.

[29] Penarrubia J, Fabregues F, Manau D, Creus M, Casals G, Casamitjana R, Carmona F, Vanrell JA, Balasch J (2005). Basal and stimulation day 5 anti-Mullerian hormone serum concentrations as predictors of ovarian response and pregnancy in assisted reproductive technology cycles stimulated with gonadotropin-releasing hormone agonist—gonadotropin treatment. Hum Reprod.

[30] Capalbo A, Rienzi L, Cimadomo D, Maggiulli R, Elliott T, Wright G, Nagy ZP, Ubaldi FM (2014). Correlation between standard blastocyst morphology, euploidy and implantation: an observational study in two centers involving 956 screened blastocysts. Hum Reprod.

[31] Minasi MG, Colasante A, Riccio T, Ruberti A, Casciani V, Scarselli F, Spinella F, Fiorentino F, Varricchio MT, Greco E (2016). Correlation between aneuploidy, standard morphology evaluation and morphokinetic development in 1730 biopsied blastocysts: a consecutive case series study. Hum Reprod.

[32] Lagalla C, Tarozzi N, Sciajno R, Wells D, Di Santo M, Nadalini M, Distratis V, Borini A (2017). Embryos with morphokinetic abnormalities may develop into euploid blastocysts. Reprod Biomed Online.

[33] Bonetti TC, Haddad DC, Domingues TS, Alegretti JR, Motta E, Seeley K, Silva ID (2019). Expressed proteins and activated pathways in conditioned embryo culture media from IVF patients are diverse according to infertility factors. JBRA Assist Reprod.

[34] Ohnishi Y, Totoki Y, Toyoda A, Watanabe T, Yamamoto Y, Tokunaga K, Sakaki Y, Sasaki H, Hohjoh H (2010). Small RNA class transition from siRNA/piRNA to miRNA during pre-implantation mouse development. Nucleic Acids Res.

[35] Rosenbluth EM, Shelton DN, Wells LM, Sparks AE, Van Voorhis BJ (2014). Human embryos secrete microRNAs into culture media—a potential biomarker for implantation. Fertil Steril.

[36] Wang Y, Lv Y, Gao S, Zhang Y, Sun J, Gong C, Chen X, Li G (2016). MicroRNA profiles in spontaneous decidualized menstrual endometrium and early pregnancy decidua with successfully implanted embryos. PLoS One.

[37] Hassan MQ, Gordon JA, Beloti MM, Croce CM, van Wijnen AJ, Stein JL, Stein GS, Lian JB (2010). A network connecting Runx2, SATB2, and the miR-23a~27a~24-2 cluster regulates the osteoblast differentiation program. Proc Natl Acad Sci U S A.

[38] Kropp J, Salih SM, Khatib H (2014). Expression of microRNAs in bovine and human pre-implantation embryo culture media. Front Genet.

[39] Wu L, Zhou H, Lin H, Qi J, Zhu C, Gao Z, Wang H (2012). Circulating microRNAs are elevated in plasma from severe preeclamptic pregnancies. Reproduction.

[40] Maragkakis M, Reczko M, Simossis VA, Alexiou P, Papadopoulos GL, Dalamagas T, Giannopoulos G, Goumas G, Koukis E, Kourtis K (2009). DIANA-microT web server: elucidating microRNA functions through target prediction. Nucleic Acids Res.

